# Anti-*Salmonella* and Antibiofilm Potency of *Salvia officinalis* L. Essential Oil against Antibiotic-Resistant *Salmonella enterica*

**DOI:** 10.3390/antibiotics11040489

**Published:** 2022-04-06

**Authors:** Samy Selim, Mohammed S. Almuhayawi, Hussain Alqhtani, Soad K. Al Jaouni, Fayez M. Saleh, Mona Warrad, Nashwa Hagagy

**Affiliations:** 1 Department of Clinical Laboratory Sciences, College of Applied Medical Sciences, Jouf University, Sakaka 72388, Saudi Arabia; 2 Department of Medical Microbiology and Parasitology, Faculty of Medicine, King Abdulaziz University, Jeddah 21589, Saudi Arabia; 3 Department of Clinical Pharmacy, College of Pharmacy, Najran University, Najran 55461, Saudi Arabia; hmhalqhtani@nu.edu.sa; 4 Hematology/Pediatric Oncology, Yousef Abdulatif Jameel Scientific Chair of Prophetic Medicine Application, Faculty of Medicine, King Abdulaziz University, Jeddah 21589, Saudi Arabia; saljaouni@kau.edu.sa; 5 Department of Medical Microbiology, Faculty of Medicine, University of Tabuk, Tabuk 71491, Saudi Arabia; fsaleh@ut.edu.sa; 6 Department of Clinical Laboratory Sciences, College of Applied Medical Sciences at Al-Quriat, Jouf University, Al-Quriat 77454, Saudi Arabia; mfwarad@ju.edu.sa; 7 Department of Biology, College of Science and Arts at Khulis, University of Jeddah, Jeddah 21959, Saudi Arabia; niibrahem@uj.edu.sa; 8 Botany and Microbiology Department, Faculty of Science, Suez Canal University, Ismailia 41522, Egypt

**Keywords:** *Salvia officinalis*, antimicrobial activity, antibiofilm, antibiotic resistant, food, *Salmonella enterica*, raw milk

## Abstract

Raw milk is a significant vehicle for the transmission of different infections. In the present study, we focused on *Salmonella enterica* from raw milk and its resistance to various antibacterial drugs. Furthermore, we have investigated the antimicrobial and antibiofilm effects of essential oil (EO) obtained from *Salvia officinalis* L. leaves that were collected from the Aljouf region, Saudi Arabia, against *S. enterica*. One-dozen strains of *S. enterica* were found in a batch of a hundred milk samples, and those *S. enterica* strains were shown to be resistant to several antibiotics, particularly the β-lactam group of antimicrobial drugs. Against multidrug-resistant *S. enterica*, the inhibitory zones for EO from *S. officinalis* leaves were found to be 21 mm in diameter. *S. officinalis* EO at 5% concentration showed a remarkable in vitro inhibitory activity toward the biofilm growth of different *S. enterica* isolates. Analysis of EO by GC–MS identified 21 distinct components, accounting for 89.94% of the total oil component. The most prominent compounds were 1,8-cineole (39.18%), β-caryophyllene (12.8%), and α--terpineol (10.3%). Taken together, our results unequivocally confirm that the *S. officinalis* EOs exert numerous bioactivities. Thus, the well-deserved attention on *S. officinalis* EO usage as a food preservative and adjunctive remedy for bacterial food-borne diseases is justified.

## 1. Introduction

Several bacteria live and thrive in milk, making it a potentially dangerous food source [1]. Toxic bacteria in milk can be transmitted by direct contact with contaminated sources on dairy farms and discharge from diseased animals’ udders [2]. The present controversy over the production of dairy products from unpasteurized milk directs current research to once again highlight the cost of consumption of such products on human health and to look for additional ways to make dairy products safer to use [2]. It is no secret that dairy farms are a major source of food-borne diseases [1]. Because most food-borne infections live in the ruminant digestive system, milking cows are an obvious source of *Salmonella*, *Campylobacter*, and *E. coli* that produce the Shiga toxin. Various studies have shown that the frequency of *Salmonella* species in bulk tank milk can vary from one percent to over 9%, and *Salmonella* species have been related to sickness in both animals and people, with the human food-borne disease as the common complaint [3,4,5,6].

Over 91,000 cases of *Salmonella enterica*-related salmonellosis have been reported in the EU so far this year, making it the second most common bacterial food-borne disease in Europe [7]. There are approximately 2600 different serotypes of *S. enterica*, a Gram-negative bacteria, and these may be divided into two major categories based on their pathogenicity: typhoidal and nontyphoidal [8,9]. Typhoidal *Salmonella* is the most common cause of human mortality; on the other hand, nontyphoidal *Salmonella* infections are self-limiting and do not require antibiotic treatment [10,11]. An essential part of treating a serious disease involves the utilization of safe antimicrobial medicines, such as fluoroquinolones and third-generation cephalosporin antibiotics that may be prescribed for both adults and children [12]. Human health may be harmed more by infection with multidrug-resistant *Salmonella* than by less-resistant bacteria. In the 21st century, antibiotic resistance is a serious public health concern, according to the World Health Organization (WHO). Antibiotic-resistant microorganisms are responsible for the deaths of more than 70,000 individuals worldwide each year, and according to predictions, this issue will soon impact millions of individuals [13]. As bacterial illnesses become more common, antibiotic resistance becomes a significant factor. Therefore, public health organizations need to practice design, implementation, and assessment to understand the degree of antibiotic resistance in different bacterial species [13]. Animals can transmit antibiotic resistance to humans either directly (through direct contact with blood or saliva), or indirectly (by consumption of infected commodities such as eggs, meat, or dairy products) [14]. Many factors contribute to the presence of antibiotic-resistant bacteria in the food supply chain, including the overuse of antibiotics in aquaculture, animal husbandry, and crop production, in addition to the transmission of antibiotic-resistant bacteria through the environment and other food sources [14,15]. According to research [16,17], antibiotic-resistant strains of *Salmonella* in humans have been linked to animals that generate food such as fish, poultry, eggs, meat, and dairy products that can all contain different *Salmonella* serovars.

Antimicrobial therapies are not recommended for self-limiting simple *Salmonella* infections in individuals without underlying comorbidities or in patients who are not immunocompromised, but they are required for invasive *Salmonella* infections [10]. Enteric fever has a 30% mortality rate if left untreated; however, it can be reduced to as low as 0.5% with adequate antibiotic therapy [9,10]. Due to the widespread emergence of strains resistant to traditional first-line antibiotics such as ampicillin, chloramphenicol, and trimethoprim, current recommendations suggest treating invasive and severe Salmonella infections with fluoroquinolones (e.g., ciprofloxacin) or extended-spectrum cephalosporins (e.g., ceftriaxone). The widespread use of fluoroquinolones, on the other hand, has been linked to a reduction in sensitivity and resistance to these medications. As a result, new antimicrobial agents were required to treat multiresistant *Salmonella* strains.

Sage, a member of the Lamiaceae family (*Salvia officinalis* L.), also known as common sage, is extensively used as a medicinal and culinary plant. Sage may be found in its natural condition as well as in extracts and EOs [18,19]. In addition to its traditional uses in food and medicine, sage includes many compounds with a wide range of intriguing characteristics. Its biological characteristics, including anti-inflammatory, antibiotic, fungistatic, virostatic, astringent, eupeptic, and antihydrotic, have an impact on human health [20,21,22,23]. Their hypoglycemic and antimutagenic properties as well as their use in treating Alzheimer’s disease, may make them useful in the pharmaceutical and medical industries [24,25]. It is popular in the pharmaceutical and cosmetics industries to employ EOs which are also used in food preparation and aromatherapy [26,27,28]. Sage EOs are combinations of a number of different active components. Sage EO chemical composition has been researched in several areas across the world, including Tunisia, Greece, and Algeria as well as the Balkans, Iran, the Middle East (Kashmir), Turkey, Croatia (Croatia), and Saudi Arabia (Saudi Arabia) [29,30,31,32]. Since these aspects are so important, researchers have investigated sage EO’s chemical composition and biological activity [28,33,34,35]. Although Saudi Arabia is a desirable location for sage cultivation because of its large botanical variety, all available information on the herb comes from sources outside Saudi Arabia [36]. Therefore, the objective of the current investigation was to detect *S. enterica* in the raw milk of cows and to study the biological activities of the EO of the leaves of the common sage, *S. officinalis* L. obtained from the Aljouf region in Saudi Arabia on the *S. enterica* strains that demonstrated resistance to several antibiotics.

## 2. Materials and Methods

### 2.1. Chemicals

All solvents (DMSO and methanol) and drugs (ampicillin, chloramphenicol, cefotaxime, ciprofloxacin, gentamicin, nalidixic acid, penicillin, and oxytetracycline) were obtained from Sigma Aldrich Co. Ltd. (St. Louis, MO, USA).

### 2.2. Samples Collection and Bacterial Isolation

One hundred samples of raw cow’s milk were gathered for analysis. As soon as the microbial isolation procedure began, all samples were stored at 4 °C until needed. In total, 0.1 mL was injected as a selective pre-enrichment broth into 10 mL of Rappaport-Vasiliadis and selenite cystine broth. Before enrichment, 1 mL of the broth was subcultured into the medium of the xylose lysine deoxycholate (XLD) and the MacConkey agar, respectively. Antisera specific to somatic, (BD Difco™ *Salmonella* O Antisera, USA) and flagella (BD Difco *Salmonella* H Antisera, Becton, NJ, USA) antigens were used to identify *S. enterica* strains according to the manufacturer’s instructions.

### 2.3. Testing for Microorganism Sensitivity

The original stock cultures of *S. enterica* were used in all tests to avoid the loss of antibiotic resistance that might occur when frequently subculturing. From the pure and fresh *S. enterica* growth, 0.5 McFarland suspensions were prepared using sterile normal saline [31]. Then, the suspension was inoculated on Müeller–Hinton agar for modified Kirby–Bauer disk-diffusion susceptibility analysis. Following the National Committee for Clinical Laboratory standards (NCCLS), *S. enterica* was tested using the disk diffusion technique. Discs of antibiotics (ampicillin (10 µg), chloramphenicol (10 µg), cefotaxime (5 µg), ciprofloxacin (10 µg), gentamicin (10 µg), nalidixic acid (10 µg), penicillin (10U), and oxytetracycline (30 µg)) were placed on the agar surface after the inoculum had dried. After 24 h of incubation at 37 °C, the diameter in mm of clear zones around the antibiotic discs suggesting bacterial growth inhibition was evaluated.

### 2.4. Collection and Identification of Plants

**Figure 1:**
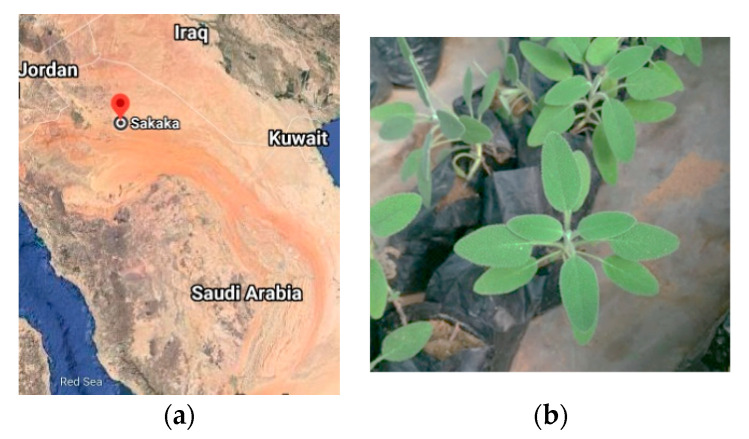
(**a**) Map of Kingdom of Saudi Arabia showing sampling site of the Sakaka, Aljouf region area and (**b**) *S. officinalis* L. leaves.

*S. officinalis* L. leaves (Figure 1) were collected from the Sakaka (29°58′11″ N 40°12′00″ E), Aljouf Botanical Gardens, maintained by the Ministry of Agriculture, Aljouf province, Kingdom of Saudi Arabia. The institutional botanist identified the plants and confirmed them by Dr. Ahmed Elbanhawy as *Salvia officinalis* L. At least five representative samples have been vouched and deposited at SCUI herbarium under the accession numbers Am_18012021/5. As a result, no permits were needed to gather the plant because it grew wild in public gardens and the desert and was not listed as endangered or protected. For 7 days, the samples were air-dried in the shade at room temperature.

### 2.5. Extraction of Salvia Essential Oil (SEO)

Hydrodistillation is used to extract essential oils from *S.* *officinalis* L. leaves using a Clevenger-type apparatus. A glass flask (2 L) is filled with 100 g of leaves material and 1500 mL of water, then shaken. After the first drop of distillate is seen at the condenser’s exit, the extraction time is recorded and the distillation completed. Calcium chloride is used to dry the oil and keep it cool (4 °C) in a firmly sealed, opaque glass flask [31]. The following equation pronounces the essential oil yield.

$$Essential\;oil\;yield\;\left(\%\right)=\frac{Weight\;of\;extracted\;oil\times 100}{Weight\;of\;plant\;materials}$$

### 2.6. GC and GC-MS Analysis Conditions of EO

GC analysis was performed on a Hewlett Packard 5890 II gas chromatograph equipped with an FID and HP-5 MS capillary column (bonded and cross-linked 5% phenyl-methylpolysiloxane 30 m × 0.25 mm, (film thickness 0.25 mL). Injector and detector temperatures were set at 220 and 290 °C, respectively. The oven temperature was held at 50 °C for 3 min, then programmed to 240 °C at a rate of 3 °C/min. Helium was the carrier gas at a flow rate of 1 mL/min. Diluted samples (1/100 in acetone, *v*/*v*) of 1.0 lL were injected manually and in the splitless mode. Quantitative data were obtained electronically from FID area percent data. GC-MS analysis of the EO was performed under the same conditions with GC (column, oven temperature, flow rate of the carrier gas) using a Hewlett Packard 5890 II gas chromatograph equipped with a Hewlett Packard 5972 mass selective detector in the electron impact mode (70 eV). Injector and MS transfer line temperatures were set at 220 and 290 °C, respectively. The components were identified based on the comparison of their relative retention time and mass spectra with those of standards, NBS75K library data of the GC-MS system, and literature data [37,38]. Alkanes were used as standards in calculating relative retention indices (RRI).

### 2.7. Anti-Salmonella Activity and Minimum Inhibitory Concentration (MIC) Assay

A well diffusion approach was used to assess the anti-*Salmonella* activity of EO. Spreading 100 μL of the 10^8^ CFU mL^–1^ test culture with a sterile glass spreader inoculates the agar plate surface. The culture was allowed to dry for 3–5 min. A sterile cork borer was used to make 5 mm-diameter holes on the agar’s surface. The plates were incubated at 28 °C for 48 h with 50 μL of 30 mg mL^–1^ of each sage oil solution in each well. The inhibitory zone’s diameter was measured in millimeters after incubation. It was necessary to do the test five times. There was a positive control, which was ampicillin, and sterile water as a negative control. The MIC was determined by preparing a range of EO strengths (0 to 5%) by dissolving in DMSO and then injecting into Müeller–Hinton agar wells (8 mm in diameter). There was 2 h of cooling in the fridge, then 24 h of incubation at 37 °C. For recording the inhibition zones to actual concentrations of EO, Vernier calipers were used.

### 2.8. In Vitro Time-Kill Curve of EO on the Growth of Salmonella

The *Salmonella* strain was inoculated in XLD broth with 5% *v*/*v* of EO and incubated at 37 °C [21]. The bacterial burden was assessed regularly until the growth curve of the control samples reached the stationary phase. As part of the analysis, a series of decimal dilutions of peptone water were prepared, and then 100 µL of each was pour-plated onto XLD (Oxiod, UK). Each plate was kept at 37 °C for 24 h. Each test was carried out in three separate sessions and valued as CFU/mL.

### 2.9. Antibiofilm Tests

The impact of EO on *Salmonella* biofilm development was qualitatively assessed as described Xiao et al. [39]. In 96-well cell culture plates, 40 µL of exponentially developing cells was dispersed. The wells were incubated for 24 h at 37 °C with the addition of 5% *v*/*v* EO. Unattached cells were removed from the solution and rinsed with Phosphate Buffer Saline after incubation (PBS). Once it had cured, the plate was dyed with 0.1% (*v*/*w*) crystal violet (Sigma-Aldrich, Darmstadt, Germany). Each test was performed in three separated replica.

### 2.10. Statistical Analysis

In order to perform all assessments, we used SPSS version 20.0 to import the antibacterial effect statistics from the study on sage.

## 3. Results

### 3.1. Drug Resistance of S. enterica Isolates toward Antimicrobial Drugs

**Figure 2:**
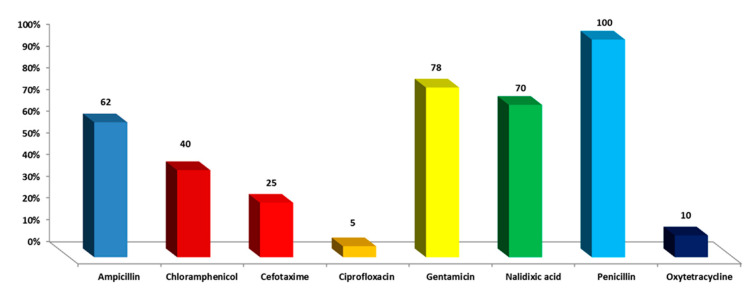
Comparison of drug resistance patterns of S. *enterica* isolates against studied antimicrobial agents.

Out of 100 raw milk samples tested, 12 strains of *S. enterica* were found, representing a 12.5% prevalence. Resistance to penicillin was 100%, and gentamicin resistance was 78% in the isolates that were recovered. As shown in Figure 2, resistance to nalidixic acid was 70%, ampicillin 62%, and chloramphenicol 40%.

### 3.2. Antibacterial Activity of S. officinalis L. EO on S. enterica Isolates

**Figure 3:**
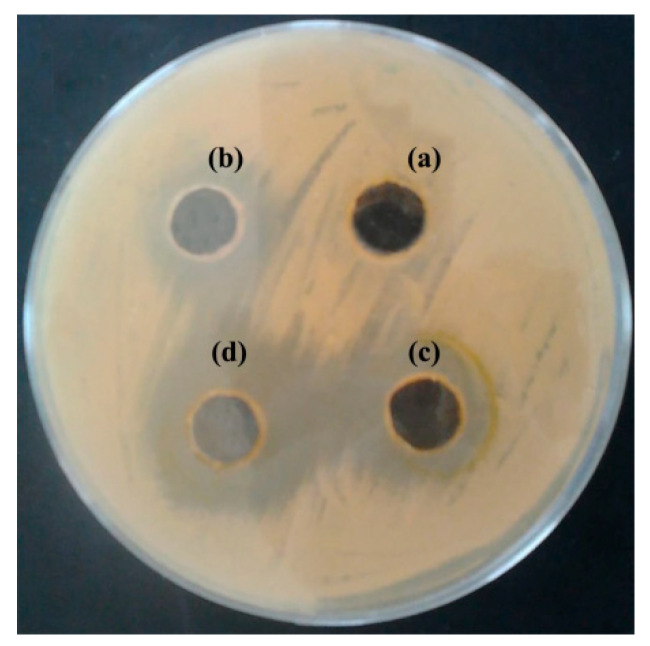
The inhibition zone of essential oil *of S. officinalis* L. leaves against *S. enterica*, at a concentration of (**a**) 0% represents the negative control, (**b**) 1% *v*/*v* (**c**) 2% *v*/*v,* and (**d**) 5% *v*/*v* in dimethylsulfoxide (DMSO).

The MIC of the EO were engaged using the well diffusion technique to assess their bacteriostatic and bactericidal capabilities. The levels of the effective EO were reported in Table 1 and depicted in Figure 3. The inhibitory action of EO of *S. officinalis* began at 1% *v*/*v* with inhibition zones of 9 mm against *S. enterica* whereas inhibited bacterial growth of these strains at concentration of 5% *v*/*v* with inhibition zones of 21 mm.

### 3.3. Effect of S. officinalis L. EO on Viability of S. enterica

**Figure 4:**
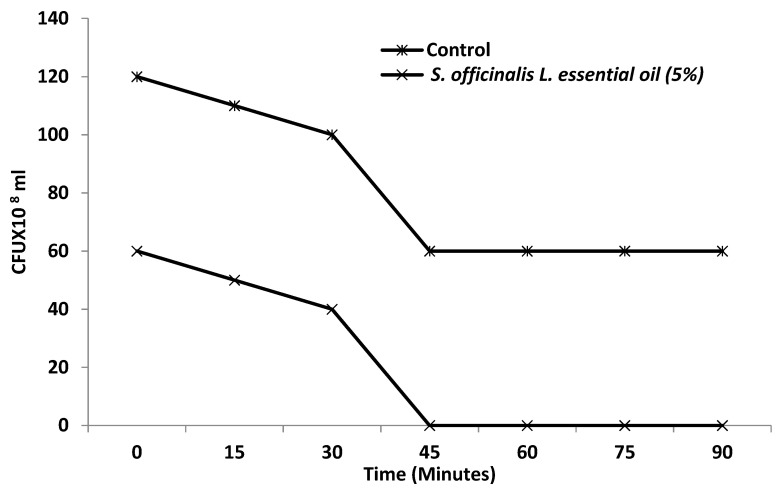
Effect of EO of S. *officinalis* at 5% *v*/*v* concentration on the viability of *S.* *enterica*. Values are the average of three individual replicates (means ± S.D.).

Based on these results, we have focused our efforts to investigate the inhibitory potency of the EO on viability and biofilm formation of *S. enterica*. Toward this, *S. officinalis* L. EO at 5% concentration has a possible antibacterial impact on the viability of *S. enterica* cells, as evidenced by the influence on cell viability. The cell viability of *S. enterica* was completely inhibited by *S. officinalis* L. EO exposure durations of 5% at 45 min (Figure 4).

### 3.4. Effect of S. officinalis L. EO on Biofilm Growth of S. enterica Isolates

**Figure 5:**
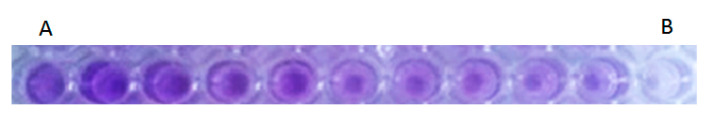
Qualitative biofilm assessment of *S. enterica* by 96-well microtiter plate evaluating (**A**) the point of wells with biofilm establishment and (**B**) specifies wells with no biofilm formation in the treatment by *S. officinalis* L. EO (5% *v*/*v*).

Biofilms formed by twelve *S. enterica* isolates were removed after one hour of contact to 5% EO from *S. officinalis* L. (Figure 5).

### 3.5. Chemical Characterization of S. officinalis L. EO by GC

The EO obtained from a sage was analyzed for the presence, in percentages, of their constituents. The experimental findings demonstrate that a particle size of 2 mm, a condensation flow rate of 1.4 mL/min, and an extraction period of 180 min resulted in a high yield of 0.8%. The identified compounds, qualitative and quantitative analytical results by GC and GC/MS are shown in Table 2, according to their elution order on a HP-5 MS capillary column. The GC–MS analysis of the EO led to the identification of 20 different components, representing 89.94% of total oil constituents (Table 2). A total of 21 constituents representing 89.94% of the oil were identified: 1,8-cineole (39.18%), β-caryophyllene (12.8%), α-terpineol (10.32%), α-humulene (7.37%), and carvone (4.25%) were the main components of the oil. A portion (0.23%) of the total composition was not identified.

## 4. Discussion

One of the most common food-borne illnesses is caused by the bacterium *S. enterica* [40]. Studies have shown that antimicrobial resistance to β-lactams, aminoglycosides, and tetracyclines is higher, while resistance to ciprofloxacin is lower [41]. Another study found a similar result for β-lactam and macrolide antibiotics (52.9–100%), while resistance to chloramphenicol was lower (3.13%), and higher resistance to ciprofloxacin and β-lactams (68.75%), tetracycline (65.62%), and colistin sulphate (46.87) was reported [36]. Recent research suggests that increased resistance to β-lactams, tetracyclines, and fluoroquinolones may be due in part to the widespread use of antibiotics without proper supervision.

Detailed data on the chemical analysis of sage EO are available from a variety of different sources [42]. The chemotypic elements of the *S. officinalis* EO were discovered to include thujone (α -thujone), 1,8-cineole, and camphor [42]. EO yields and components may be affected by a variety of factors, including seasonal fluctuations, geographic location variances in oil quality and yields, EO extraction processes, and EOs acquired via various drying methods [43,44,45]. According to the aforementioned factors, significant variability in sage EO components have been observed. When dried in an oven at 45 or 65 °C, as well as in the microwave oven (500 W), the sage EO’s chemotypic products, 1,8-cineole and camphor, considerably differed from batch to batch [46]. EO batches derived from plants harvested at various times of the year and from different geographic regions included varying amounts of α -thujone and camphor [47].

It has been speculated that the EOs of *S. officinalis* might contain beneficial substances, and as previously stated, the sage EO has antimicrobial properties, supported by this study [48]. Therefore, the antibacterial properties of sage EO can be attributed to certain oil components, including camphor, 1,8-cineole, and α-pinene, all of which are found in high concentrations [49]. These substances are well known for their antibacterial action against many microbes [50]. According to previous research, phenolic compounds have also been linked to antibacterial activity [51]. The results show that this natural substance may be used in various sectors, including food preservation and medical treatment [52]. Its antimicrobial activity is affected by the variability of the chemical composition of *S. officinalis* EOs. This oil depends on several factors (such as genetic background, region, environmental conditions, season, plant parts used for extraction, and the extraction method) [53,54]. To fully understand Saudi *S. officinalis* L. and its active constituents in antimicrobial pharmaceuticals both locally and systemically, further research is required.

## 5. Conclusions

Providing a safe and nutrient-dense food supply is challenging due to several variables involved from farm to fork. There is a clear connection between food-borne pathogens such as *S. enterica* and dairy food safety. In the current study, we have investigated the inhibitory activity of *S. officinalis*. L. extract and EO on *S. enterica*. A selective inhibition against potentially pathogenic *Salmonella* and a significant inhibition of the biofilm formation were shown by the *S. officinalis* L. EO. To our knowledge, this is the first report on the characterization of *S. officinalis* EO for its antibiofilm properties against *Salmonella*. According to our findings, *S. officinalis* L. EO gathered in the Aljouf region, Saudi Arabia, may be used in the future for the development of antibacterial drugs against multidrug-resistant *S. enterica*.

## Figures and Tables

**Table 1:** Inhibition zone diameter and MIC of *S. officinalis* L. EO against *S.* *enterica*.

Item	*Salmonella enterica*			
	Inhibition Zone Diameter (mm)	MIC (µg/mL)		
		MIC	MIC_50_	MIC_90_
**Essential Oil ^a^**	21 ± 1.09	1	2	5
**DMSO (Negative control)**	-	-	-	-

^a^ Concentration 5% *v*/*v* EO in DMSO. Sensitive (Inhibition Zone Diameter ≥11 mm: Bauer et al. [31]). Results are Mean ± S.D. of triplicate experiments.

**Table 2:** Chemical composition of *S. officinalis* L. EO.

Peak No	Compound ^a^	RI ^b^	KI ^c^	Composition (%)
**1**	α-Pinene	938	940	0.74
**2**	Camphene	954	954	0.86
**3**	Myrcene	994	992	3.36
**4**	α-Terpinene	1018	1019	0.18
**5**	p-Cymene	1026	1025	0.72
**6**	Limonene	1030	1030	0.42
**7**	1,8-Cineol	1035	1033	39.18
**8**	β-Ocimene	1040	1041	0.14
**9**	γ-Terpinene	1062	1063	0.12
**10**	α-Terpinolene	1090	1089	0.09
**11**	Linalool	1098	1098	0.99
**12**	Borneol	1068	1170	0.36
**13**	α-Terpineol	1188	1189	10.32
**14**	Myrtenol	1193	1194	3.13
**15**	Carvone	1246	1248	4.25
**16**	Bornyl acetate	1285	1286	0.15
**17**	β-Caryophyllene	1419	1419	12.8
**18**	α-humulene	1455	1456	7.37
**19**	Germacrene-B	1535	1536	0.38
**20**	Viridiflorol	1591	1591	4.15
**21**	UD ^e^	1695	1695	0.23
Identified components (%)		89.94		
Monoterpene hydrocarbons		6.28		
Oxygenated monoterpenes		55.73		
Sesquiterpene hydrocarbons		20.55		
Oxygenated sesquiterpenes		4.15		
**Oil yield (%) (*v*/*w*)** 0.45				

^a^ Components were identified through KI and GC–MS (gas chromatograph coupled with mass spectrometry) and listed according to their elution on HP-5 MS capillary column (30 m). ^b^ RI: Retention Index. ^c^ KI: Kovats indexes on HP-Innowax capillary column in reference to C9-C28 n-alkanes [37,40]. ^d^ UD: not identified compound.

## Data Availability

Data is contained within the article.
